# Supplementary figures and images for: Impact of the entorhinal feed-forward connection to the CA3 on hippocampal coding
Source: PLoS One. 2025 Jul 17;20(7):e0326032. doi: 10.1371/journal.pone.0326032 (PMC12270128; doi:10.1371/journal.pone.0326032)

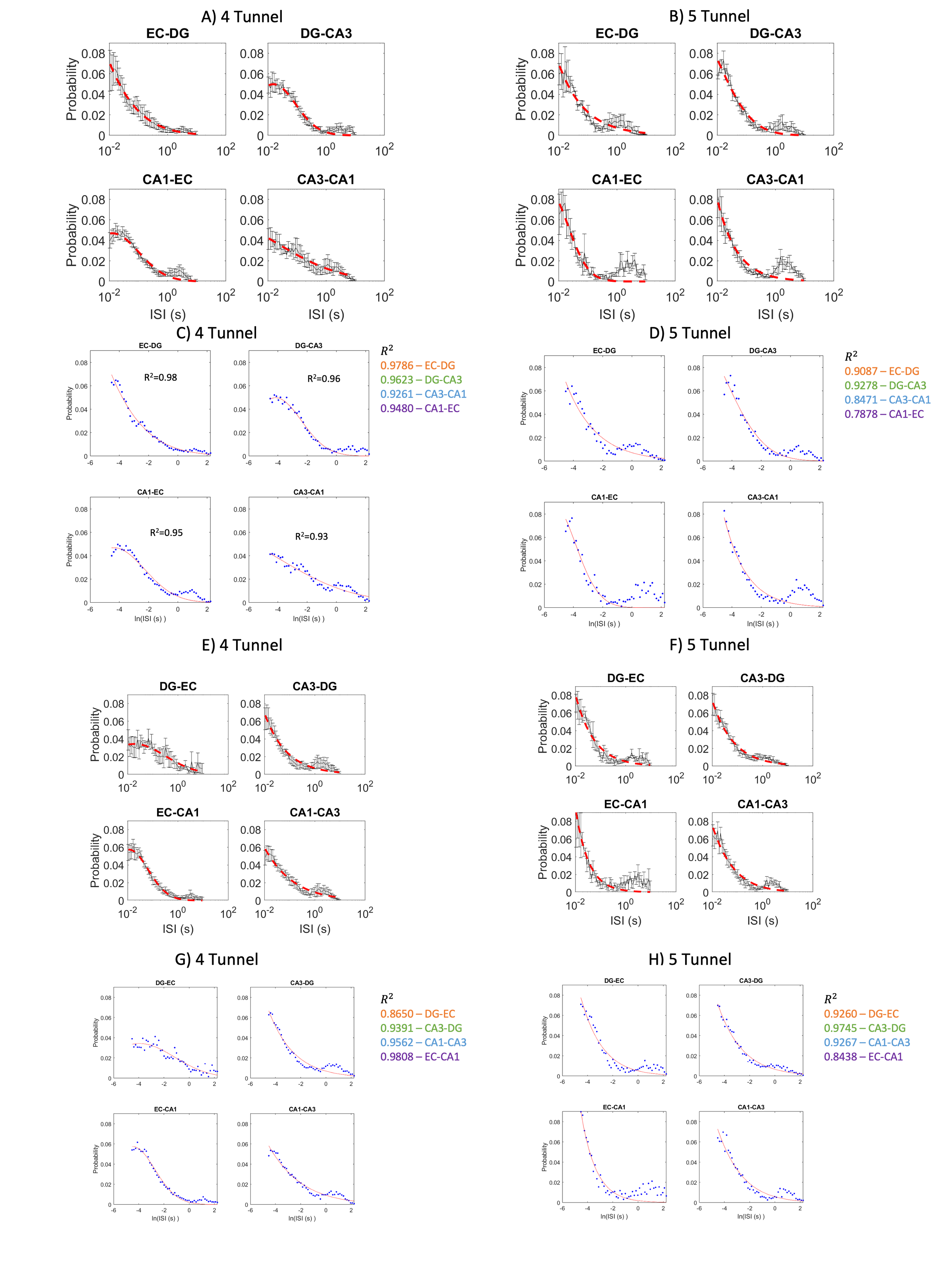

Supplement: S1 Fig — Both four-tunnel and five-tunnel axon architectures show non-Gaussian semi-log distributions of ISIs. (A–D) feed-forward (FF). (E–H) feedback (FB). (TIF) [file pone.0326032.s001.tif]

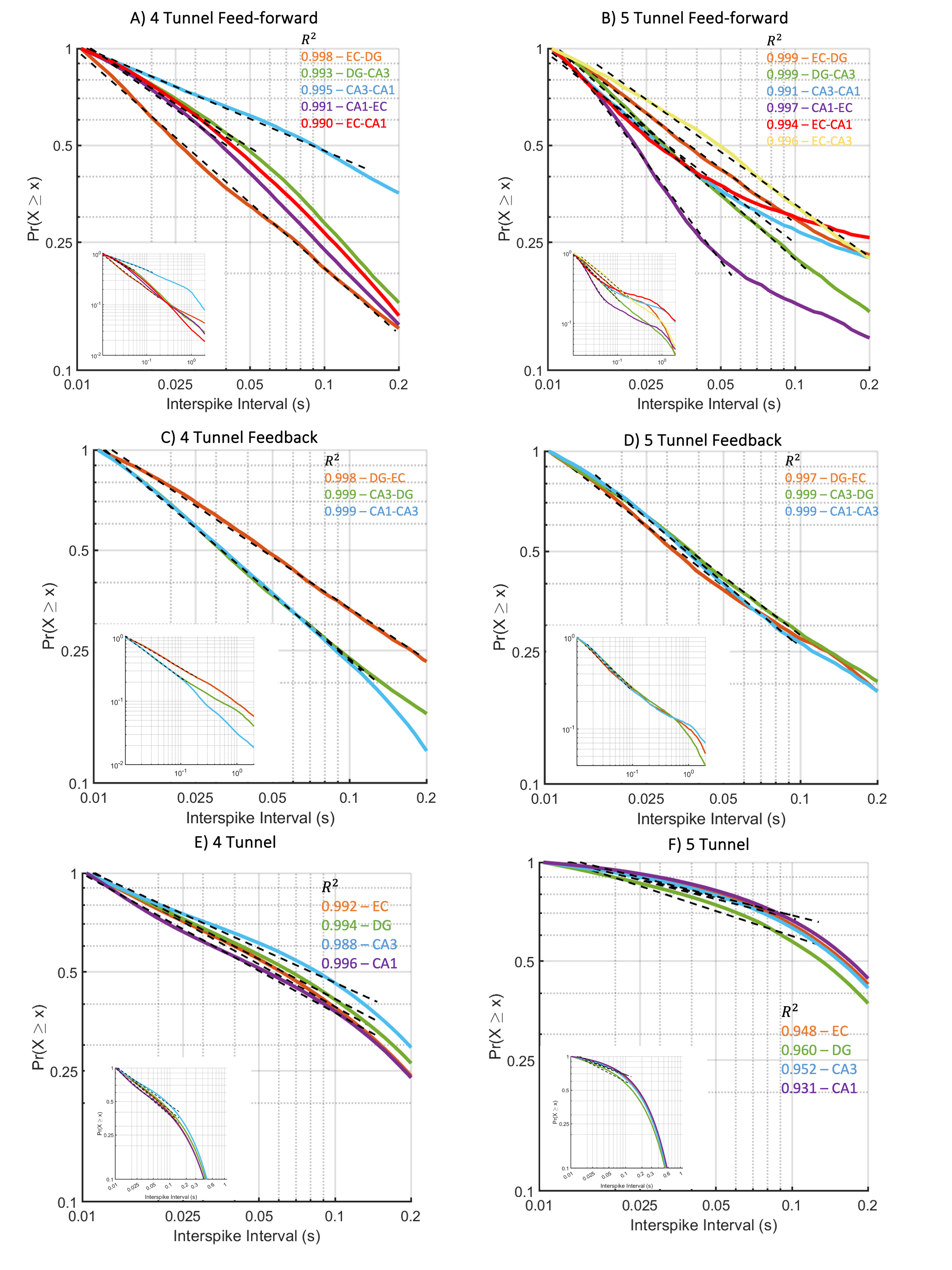

Supplement: S2 Fig — (A and B) axonal feed-forward (FF). (C and D) axonal feedback (FB). (E and F) subregional. (TIF) [file pone.0326032.s002.tif]
