# Supplementary material for: Impact of the entorhinal feed-forward connection to the CA3 on hippocampal coding
Source: PLoS One. 2025 Jul 17;20(7):e0326032. doi: 10.1371/journal.pone.0326032 (PMC12270128; doi:10.1371/journal.pone.0326032)
Supplement: Attachment — Submitted filename: PONE-D-24-49403_reviewer.pdf [file pone.0326032.s003.pdf]

PLOS ONE

Impact of the entorhinal feed-forward connection to the CA3 on hippocampal coding

--Manuscript Draft--

**Manuscript Number:**	PONE-D-24-49403
**Article Type:**	Research Article
**Full Title:**	Impact of the entorhinal feed-forward connection to the CA3 on hippocampal coding
**Short Title:**	Impact of the entorhinal feed-forward connection to the CA3 on hippocampal coding
**Corresponding Author:**	Gregory J Brewer, PhD<br>University of California Irvine<br>Irvine, CA UNITED STATES OF AMERICA
**Keywords:**	Hippocampus; memory; Learning; Multielectrode array, EC; DG; CA3; CA1; Perforant Pathway; Neuron; Neural Networks; Axons; Microfluidics; Spiking Dynamics; Burst Dynamics; Burstiness; Power law Distributions; Cell Culture
**Abstract:**	Each sub-region of the hippocampus plays a critical computational role in the formation of episodic learning and memory, but studies have yet to show and interpret the individual spiking dynamics of each region and how that information is passed between each subregion. This is in part due to the difficulty in accessing individual communicating axons. Here, we created a novel microfluidic device that facilitates network growth of four separated hippocampal subregions over a micro-electrode array. This device enabled monitoring single axons over two electrodes so direction of spike propagation in interregional communication could be ascertained. In this in vitro hippocampal study, we compared spiking dynamics across two novel four-compartment device architectures: one with four sets of axon tunnels between subregions that excluded the perforant pathway from EC-CA3, and one with five sets of axon tunnels that included the EC-CA3 connection. We found 30-90% faster feed-forward firing rates (shorter interspike intervals) in axons in the five-tunnel model with 35-75% slower bursting dynamics (longer interburst intervals) compared to the four-tunnel model. Comparing the percentage of spikes in bursts between array designs, the five-tunnel architecture showed that the CA3-CA1 and CA1-EC axons had more spikes in bursts than the four-tunnel counterpart suggesting more structured information transfer. Feedback firing rates were similar between configurations. The faster feed-forward inter-regional spiking in the more natural five-tunnel configuration than with four-tunnel suggests tighter control of spiking and possibly more precise communication between subregions.
**Order of Authors:**	Samuel Brandon Lassers, B.S.
	Shazfa Khatri
	Ruiyi Chen, Ph.D
	Yash Vakilna, M.S.
	William Tang, Ph.D
	Gregory Brewer, Ph.D
**Additional Information:**	
**Question**	**Response**
**Financial Disclosure**<br><br>Enter a financial disclosure statement that describes the sources of funding for the work included in this submission. Review the submission guidelines for detailed requirements. View published research articles from *PLOS ONE* for specific	Yes

Powered by Editorial Manager® and ProduXion Manager® from Aries Systems Corporation

examples.<br><br>This statement is required for submission and **will appear in the published article** if the submission is accepted. Please make sure it is accurate.<br><br>**Funded studies**<br>Enter a statement with the following details:<br>• Initials of the authors who received each award<br>• Grant numbers awarded to each author<br>• The full name of each funder<br>• URL of each funder website<br>• Did the sponsors or funders play any role in the study design, data collection and analysis, decision to publish, or preparation of the manuscript?<br><br>Did you receive funding for this work?	
Please add funding details.<br>as follow-up to "**Financial Disclosure**<br><br>Enter a financial disclosure statement that describes the sources of funding for the work included in this submission. Review the submission guidelines for detailed requirements. View published research articles from *PLOS ONE* for specific examples.<br><br>This statement is required for submission and **will appear in the published article** if the submission is accepted. Please make sure it is accurate.<br><br>**Funded studies**<br>Enter a statement with the following details:<br>• Initials of the authors who received each award<br>• Grant numbers awarded to each author<br>• The full name of each funder<br>• URL of each funder website<br>• Did the sponsors or funders play any role in the study design, data collection and analysis, decision to publish, or preparation of the manuscript?	The author(s) declare financial support was received for the research, authorship, and/or publication of this article. This study was supported in part by funds from the UC Irvine Foundation. The Foundation had no decision making capacity for the design or manuscript.

Did you receive funding for this work?"	
Please select the country of your main research funder (please select carefully as in some cases this is used in fee calculation).<br>as follow-up to "**Financial Disclosure**<br><br>Enter a financial disclosure statement that describes the sources of funding for the work included in this submission. Review the submission guidelines for detailed requirements. View published research articles from *PLOS ONE* for specific examples.<br><br>This statement is required for submission and **will appear in the published article** if the submission is accepted. Please make sure it is accurate.<br><br>**Funded studies**<br>Enter a statement with the following details:<br>• Initials of the authors who received each award<br>• Grant numbers awarded to each author<br>• The full name of each funder<br>• URL of each funder website<br>• Did the sponsors or funders play any role in the study design, data collection and analysis, decision to publish, or preparation of the manuscript?<br><br>Did you receive funding for this work?"	UNITED STATES - US
**Competing Interests**<br><br>Use the instructions below to enter a competing interest statement for this submission. On behalf of all authors, disclose any competing interests that could be perceived to bias this work—acknowledging all financial support and any other relevant financial or non-financial competing interests.	The authors declare that the research was conducted in the absence of any commercial or financial relationships that could be construed as a potential conflict of interest.

Powered by Editorial Manager® and ProduXion Manager® from Aries Systems Corporation

This statement is required for submission and **will appear in the published article** if the submission is accepted. Please make sure it is accurate and that any funding sources listed in your Funding Information later in the submission form are also declared in your Financial Disclosure statement.<br><br>View published research articles from *PLOS ONE* for specific examples.<br><br>**NO authors have competing interests**<br><br>Enter: *The authors have declared that no competing interests exist.*<br><br>**Authors with competing interests**<br><br>Enter competing interest details beginning with this statement:<br><br>*I have read the journal's policy and the authors of this manuscript have the following competing interests: [insert competing interests here]*<br><br>* typeset	
**Ethics Statement**<br><br>Enter an ethics statement for this submission. This statement is required if the study involved:<br><br>• Human participants<br>• Human specimens or tissue<br>• Vertebrate animals or cephalopods<br>• Vertebrate embryos or tissues<br>• Field research<br><br>Write "N/A" if the submission does not require an ethics statement.<br><br>General guidance is provided below. Consult the submission guidelines for detailed instructions. **Make sure that all information entered here is included in the Methods section of the manuscript.**	The animal study was approved by UC Irvine Institutional Animal Care and Use Committee. The study was conducted in accordance with the local legislation and institutional requirements.

**Format for specific study types**<br><br>**Human Subject Research (involving human participants and/or tissue)**<br>• Give the name of the institutional review board or ethics committee that approved the study<br>• Include the approval number and/or a statement indicating approval of this research<br>• Indicate the form of consent obtained (written/oral) or the reason that consent was not obtained (e.g. the data were analyzed anonymously)<br><br>**Animal Research (involving vertebrate animals, embryos or tissues)**<br>• Provide the name of the Institutional Animal Care and Use Committee (IACUC) or other relevant ethics board that reviewed the study protocol, and indicate whether they approved this research or granted a formal waiver of ethical approval<br>• Include an approval number if one was obtained<br>• If the study involved *non-human primates*, add *additional details* about animal welfare and steps taken to ameliorate suffering<br>• If anesthesia, euthanasia, or any kind of animal sacrifice is part of the study, include briefly which substances and/or methods were applied<br><br>**Field Research**<br><br>Include the following details if this study involves the collection of plant, animal, or other materials from a natural setting:<br>• Field permit number<br>• Name of the institution or relevant body that granted permission	
**Data Availability**<br><br>Authors are required to make all data underlying the findings described fully available, without restriction, and from the time of publication. PLOS allows rare exceptions to address legal and ethical concerns. See the PLOS Data Policy and FAQ for detailed information.	Yes - all data are fully available without restriction

Powered by Editorial Manager® and ProduXion Manager® from Aries Systems Corporation

A Data Availability Statement describing where the data can be found is required at submission. Your answers to this question constitute the Data Availability Statement and **will be published in the article**, if accepted.<br><br>**Important:** Stating 'data available on request from the author' is not sufficient. If your data are only available upon request, select 'No' for the first question and explain your exceptional situation in the text box.<br><br>Do the authors confirm that all data underlying the findings described in their manuscript are fully available without restriction?	
**Describe where the data may be found in full sentences. If you are copying our sample text, replace any instances of XXX with the appropriate details.**<br><br>• If the data are **held or will be held in a public repository**, include URLs, accession numbers or DOIs. If this information will only be available after acceptance, indicate this by ticking the box below. For example: *All XXX files are available from the XXX database (accession number(s) XXX, XXX.*).<br>• If the data are all contained **within the manuscript and/or Supporting Information files**, enter the following: *All relevant data are within the manuscript and its Supporting Information files.*<br>• If neither of these applies but you are able to provide **details of access elsewhere**, with or without limitations, please do so. For example:<br><br>*Data cannot be shared publicly because of [XXX]. Data are available from the XXX Institutional Data Access / Ethics Committee (contact via XXX) for researchers who meet the criteria for access to confidential data.*<br><br>*The data underlying the results presented in the study are available*	The code and data are available on GitHub at https://github.com/Brewer-Neurolab/Khatri_Lassers_EC_CA3_Pathway_Effect.

from (include the name of the third party and contact information or URL).<br>• This text is appropriate if the data are owned by a third party and authors do not have permission to share the data.<br><br>* typeset	
Additional data availability information:	

Manuscript

Click here to access/download;Manuscript;FINAL Khatri 4v5 Tunnel Manuscript 241031gb2.docx

Impact of the entorhinal feed-forward connection to the CA3 on hippocampal coding

Samuel B. Lassers[1]*, Shazfa S. Khatri[1]*, Ruiyi Chen[1], Yash Shashank Vakilna[1,2], William C. Tang[1], and Gregory J. Brewer[1,3†]

[1]Department of Biomedical Engineering, University of California Irvine, Irvine, CA, United States,

[2]Texas Institute of Restorative Neurotechnologies (TIRN), The University of Texas Health Science Center

(UTHealth), Houston, TX, United States,

[3]Memory Impairments and Neurological Disorders (MIND) Institute, Center for

Neuroscience of Learning and Memory, University of California Irvine, Irvine, CA, United States

***These authors contributed equally.**

[†]Correspondence: Greg Brewer, GJBrewer@uci.edu

Abstract

Each sub-region of the hippocampus plays a critical computational role in the formation of episodic learning and memory, but studies have yet to show and interpret the individual spiking dynamics of each region and how that information is passed between each subregion. This is in part due to the difficulty in accessing individual communicating axons. Here, we created a novel microfluidic device that facilitates network growth of four separated hippocampal subregions over a micro-electrode array. This device enabled monitoring single axons over two electrodes so direction of spike propagation in interregional communication could be ascertained. In this *in vitro* hippocampal study, we compared spiking dynamics across two novel four-compartment device architectures: one with four sets of axon tunnels between subregions that excluded the perforant pathway from EC-CA3, and one with five sets of axon tunnels that included the EC-CA3 connection. We found 30-90% faster feed-forward firing rates (shorter interspike intervals) in axons in the five-tunnel model with 35-75% slower bursting dynamics (longer interburst intervals) compared to the four-tunnel model. Comparing the percentage of spikes in bursts between array designs, the five-tunnel architecture showed that the CA3-CA1 and CA1-EC axons had more spikes in bursts than the four-tunnel counterpart suggesting more structured information transfer. Feedback firing rates were similar between configurations. The faster feed-forward inter-regional spiking in the more natural five-tunnel configuration than with four-tunnel suggests tighter control of spiking and possibly more precise communication between subregions.

Introduction

The flow of information through the hippocampal trisynaptic circuit enables learning and the formation of episodic memory. The anatomical structure of this pathway, initially detailed by Ramon y Cajal in 1911, reveals a sequential relay of connections among its three regions, each with its own computational role. Information from other regions of the brain is integrated in the entorhinal cortex (EC), progresses to the dentate gyrus (DG), passes through CA3 and then CA1 (Andersen et al., 1971) before completing the loop by returning to the subiculum and entorhinal cortex. Later studies clearly identified the perforant path that connects the EC to CA3 and CA1 as well as DG, a feed-forward jump in connectivity of unclear function.

Current computational models of the hippocampus show that the trisynaptic loop allows episodes of memory to be recalled from partial cues, enables spatial learning through sparse synaptic connections, can complete episodes based on partial cues, associate learned responses with current events and learn novel patterns (Kesner and Rolls, 2015; de Camargo 2019). The hippocampus integrates sensory information and performs higher level computations from the prefrontal cortex, amygdala, orbitofrontal cortex, and the entorhinal cortex (EC), allowing for further computation of object and spatial representations and reward related information (Kesner and Rolls, 2015). Primary inputs to the DG from the EC via the perforant pathway are proposed to be essential for pattern separation (Kesner and Rolls, 2015). Perforant path axons also synapse as mossy fiber connections in the CA3, facilitating the learning of distinctive features. More activity in CA3 has also been shown to support discrimination between stimuli that share features (Cappaert et al., 2015). The CA3 associates diverse types of information and excels in the rapid encoding of novel information while aiding in efficient recall processes of pattern completion (Poli et al., 2017; Poli et al., 2018). The CA1 sequences and orders information coming from the CA3

and then routes information back to the EC and subiculum. While the separate functions of the subregions of the trisynaptic are beginning to be understood, a thorough understanding of effect on CA1 spiking dynamics of the CA3 inputs from DG compared to EC and DG inputs to CA3 could provide important information for modeling architecture as well as hippocampal coding functions.

In this study we examined the spiking dynamics across the hippocampus through the comparison of two novel four-compartment devices: one with four sets of axon tunnels between subregions monitored by substrate electrodes that excluded the perforant pathway from EC-CA3, and one with five sets of monitored axon tunnels that included the EC-CA3 connection. Using our microelectromechanical systems (MEMS) device, we simultaneously recorded spiking activity in the four subregions of the hippocampus and the axons connecting the sub-regions. The directionality of spike propagation was measured using electrode pairs underneath single axons between sub-regions to determine their feed forward or feed-back activity. In a previous analysis, the four-chamber, four-tunnel cultured hippocampal neural network showed spontaneous directional spatiotemporal dynamics in both the soma and axons within and between the sub-regions of the trisynaptic loop (Vakilna et al., 2021). This assisted the discovery of the architecture and flow of information transfer of the hippocampus. Additionally, the distribution of interspike intervals (ISIs) and interburst intervals (IBIs) were shown to be significantly different between sub-regions and follow a log-log distribution. In the present study, we expanded these findings by comparing these spikes and burst distributions in the monitored four-tunnel sets with those of five-tunnel set model of the hippocampus to explore how the perforant path of EC-CA3 axons might affect network dynamics.

Materials and methods

In vitro hippocampal neuronal network culture in a four-chamber device with four-way and five-way microfluidic interconnections

Microfluidic and culturing methods were previously described in more detail in Vakilna et al. (2021) and Chen et al. (2023). In short, we employed a novel four-chamber device containing microfluidic tunnels for axonal communication between chambers (**Fig 1**). Each chamber contained dissociated neurons from micro dissections of the entorhinal cortex (EC), dentate gyrus and hilus (DG), CA3, and CA1 including the subiculum from postnatal day 4 Sprague Dawley rat pups under anesthesia as approved by the UC Irvine Institutional Animal Care and Use Committee (IACUC). Brain cells were dissociated and plated at 1,000 cells/mm^2 for DG, 330 for CA3, 410 for CA1 (including subiculum), and 330 for EC, in order to reflect in vivo neural densities: EC-DG 1:3, DG-CA3 3:1, CA3-CA 11:1.25, and CA1-EC 1.25:1 (Braitenberg, 1981). Cells were in 10 µL of NbActiv4 medium (Transnetyx BrainBits, Springfield, IL; Brewer et al., 2009b) and were plated into the wells sequentially. After thirty minutes in the incubator to allow for adhesion, 0.8 mL culture medium was added. The cultures were capped with Teflon sheets (ALA Scientific, Farmingdale NY) and incubated for 21–26 days in humidified 5% CO_2 and 9% O_2 (Brewer and Cotman, 1989). Half of the medium was changed every 3-4 days. Activity was recorded on days 21-26, 2-5 days after a medium change when the networks had reached maturity. MEA120 glass multielectrode arrays (MEA) equipped with 120 30-µm-diameter electrodes spaced 200 µm served as the substrates for the culture of neuronal networks (Multichannel Systems, Reutlingen, Germany; ALA Scientific, Farmingdale, NY, USA). A custom polydimethylsiloxane (PDMS) device was aligned and attached to the MEA that separates the microelectrodes into four chambers

for each cell type. Axon-isolating tunnels connected each compartment. Each PDMS well was 9.7 mm^2 by 1-mm high. Each microfluidic tunnel was 3 μm high × 10 μm wide × 400 μm long spaced 50 μm apart.

Fig 1. Comparison of four- versus five tunnel architecture.

A) Four sets of tunnels connect EC to only DG and CA1 and CA3 to CA1. **B**) Five sets of tunnels add cross tunnels from EC to CA3. White lines are microfluidic tunnels for axonal communication between labeled subregions that confine neuronal somata that were micro-dissected from individual subregions of the postnatal hippocampus. Dark circles are 30 μm diameter electrodes used to measure voltage spikes. Inter-electrode spacing is 200 μm. Black lines are insulated connections to the amplifier. Adapted from Wang et al. (2024) and Chen et al. (2023).

Two configurations of microfluidic tunnels for axonal communication were compared (**Fig 1**). In the first configuration, each compartment was connected to the adjacent one to allow feed-forward connection according to Cajal's original understanding of the trisynaptic loop. Five of the 51 axon tunnels between each compartment were monitored with a pair of electrodes underneath. In the second configuration, two electrodes were under each of 4 of the 67 tunnels between the same subregions, but to instantiate the perforant path (Andersen et al., 1971), axonal tunnels diagonally connected the EC to the CA3 wells. Five of these 11 EC to CA3 tunnels were monitored by pairs of electrodes and two more by a single electrode (**Fig 1**). From the spike timing delay between the electrode pairs in each tunnel, we determined the direction of axonal potential propagation and categorization of action potentials as either feed-forward or feedback. In both configurations, nineteen electrodes were at the bottom of each subregion. The substrate was treated with oxygen plasma followed by coating the wells with poly-D-lysine for cell adhesion.

Finally, we placed micro dissected dissociated neurons from four-day-old rat hippocampi in the corresponding wells and cultured them in NbActive4 (Brewer 2008) for 18-22 days (Chen et al., 2023).

Multi-Electrode Array and Recording

A Multichannel Systems MEA120 1100 (Multichannel Systems, Reutlingen, Germany) amplifier was used to record spontaneous activity on the 120-electrode microarray. Spontaneous activity was analyzed using MC_Rack software at a sampling rate of 25 kHz at 37° C for 5 min, in humidified 5% CO_2, 9% O_2 (custom Airgas USA, Santa Ana, CA). Recordings were initiated several minutes after transfer from the culture incubator to the amplifier, once at least 80% of the tunnels had stable activity. Arrays with <80% active tunnels or that had poor growth in one of the compartments were rejected for recording.

Spike detection, sorting, and axonal propagation direction

Details on our axonal spike directionality algorithm were previously described in Lassers et al. (2023). Briefly, raw tunnel data sampled at 25 kHz were filtered through Wave_Clus (Chaure et al., 2018), and spike detection and clustering were computed from 5 to 50 S.D. noise and 50.1 to 500 S.D. to ensure the counting and clustering of large axonal spiking. A refractory period of 1.5 ms was specified. Any spike shapes differing by less than three standard deviations from the mean spike shape were included in a single cluster. The large, tolerated deviation was chosen to accommodate different axon-electrode coupling for a single axon on each of the two electrodes. Clustering was used to discard complex spikes from multiple axons in a microtunnel that produce overlapping spike waveforms. Similar to previous analyses of these tunnel devices that

demonstrated that ~63% of tunnels contained only one axon (Narula et al., 2017), single axons were identified by their uniform conduction velocities or spike timing delays. These timing delays were used to generate a normalized matching indexing (NMI) algorithm which was computed for every tunnel using the timing comparison made from the two electrodes spanning each tunnel (**Eq. 1)**. All tunnels were the same length of 400 μm except for the EC-CA3 diagonal tunnels that ranged from 400-600 μm. To rigorously determine which tunnels have single axons so that directionality could be determined, NMI was calculated with the equation:

$$NMI = \frac{\#\ paired\ spikes}{max(total\ \#spikes\ per\ spike\ train)} \qquad \textbf{Eq. 1}$$

Tunnels with NMI > 0.2 (20% of spikes matched between two clusters) were considered valid. This threshold was sufficient for eliminating spurious spike pair correlations during high spike rates.

A histogram of conduction times was generated with thresholding and peak prominence values provided by the MATLAB findpeaks function. Valid delay times were between 0.2 ms and 1 ms if there was a peak at sufficiently fast conduction times. Feed-forward axons were identified by positive delay times and feed-back axons by negative conduction times, the basis for our determination of the feed-forward and feedback directionality of axonal communication. In contrast, raw data from the wells were filtered through Wave_Clus and spikes were detected at the threshold of ±3.5 S.D. noise. This lower threshold than for axons better accommodated the lower signal to noise ratio in the wells. Spike clustering was not needed since single neurons were detected in >90% of cases.

Spike dynamics and probability distributions

Vakilna et al. (2021) showed that the distribution of inter-spike intervals (ISI) and inter-burst intervals (IBI) follow log–log distributions and were visualized as normalized complementary cumulative probability distributions (CCDs) with logarithmically spaced bins (Newman, 2005). A log-transformed linear model of the slope, m, and intercept c was used to fit the CCD after log transformation.

$$log_{10}(P) = m \times log_{10}(t) + c \qquad \textbf{Eq. 2}\ (from\ P = t^m c)$$

A grid search of the local maximum for $Pearson's\ R^2$ was used to find the best fits with time limits varied up to 50% with a step size of 5%. A single fit was found for all ISI CCDs over the probability range from 1 to 0.1. The four-tunnel ISI CCDS intervals were different for each tunnel, for both feed-forward and feedback axons. For the five-tunnel design, intervals were from 0.01 to 0.2 s except for CA1-EC feed-forward and feedback axons which were fit for intervals between 0.01 to 0.11 s and 0.01 to 0.09 s respectively, to account for non-linearities in the distribution. Two linear fits were calculated for all inter-burst interval (IBI) CCDs piecewise to account for the "up states" and "down states," referring to fast and slow bursting, respectively (Vakilna et al., 2021). The minimum time for the up states was used as the maximum time for the down states. We compared the distribution of ISI to a log-normal distribution instead of a log-log (**Supplementary Fig 1**) to show that the distributions were not log-normal. The burstiness of a neuronal unit was defined as the percentage of tagged spikes that appear in bursts, at least four spikes, each separated by less than 50 ms. Distributions of axonal and somal burstiness were plotted normalized by the number of arrays in each network design and statistical tests were

performed to assess changes in burstiness between designs.

Supplementary Fig 1. Axonal cumulative probability distributions of inter-spike intervals (ISI) and slopes in the four-tunnel (*n* = 9 arrays) and five tunnel architecture (*n* = 6 arrays) compared by subregions.

Both four tunnel and five tunnel axon architectures show non-gaussian semi-log distributions of ISIs. **A-D)** feed-forward (FF) **E-H)** feedback (FB).

Statistics

Data were analyzed with custom MATLAB 2023a scripts. Slopes were compared for significant statistical differences at alpha=0.05 using analysis of covariance (ANCOVA) followed Tukey's honest significant difference (HSD) test. The burstiness of the data was compared using the nonparametric Kruskal-Wallis test since the distributions of burstiness had significant deviations from the normal distribution to determine if the data came from different distributions at $p<0.05$. Data were combined and analyzed for nine separately plated networks for the four-tunnel model and six separately plated networks for the five-tunnel model.

Results

Different subregional and axonal spiking and bursting dynamics between 4 tunnel and 5 tunnel self-wired hippocampus model

To explore the impact of hippocampal architecture on spike dynamics, we designed a four-compartment device with microfluidic tunnels connecting sub-regions that promoted axonal self-

wiring and prevented dendritic infiltration (**Fig 1**). Each of the four compartments contained a sub-region of the trisynaptic loop (EC, DG, CA3, and CA1) and tunnels between each sub-region for their communication. The traditional method of studying hippocampal information encoding interprets behavior from electrical recording of only one hippocampal subregion, most commonly the CA1, which introduces biased attention to an individual part of the hippocampus. This method often ignores the differences in dynamics of information encoding in each sub-region of the hippocampus or in control of the axonal information that gets transmitted from one region to the next. However, this approach is often used because of the difficulty in accessing all regions of the hippocampus simultaneously and the impossibility of isolation of recordings from single axons during in vivo recordings. Superimposing the four-compartment device over a multi-electrode array allowed recording of spontaneous activity. Our four-compartment design enabled monitoring of subregional and between region activity in single axons. As previously demonstrated for axons communicating between two compartments of DG and CA3 (Narula et al., 2017), these micron-sized tunnels mostly contained only one or two axons each.

Spike dynamics of feed-forward and feedback axons reveal greater activity in four-tunnel vs. five-tunnel architecture

Information relayed between sub-regions of the hippocampus was analyzed through the neuronal spiking in communicating axons. We define feed-forward as EC>DG>CA3>CA1, back to EC in the trisynaptic loop and the reverse, if detected, as feedback timing of action potential propagation. Dual electrodes were used to record spike timing differences and determine the direction of spike propagation (Vakilna et al., 2021). Feed-forward activity was likely excitatory and feedback likely inhibitory. When axons in similar devices of CA3 connected to DG were

stained for the GABA synthesis enzyme, glutamic acid decarboxylase (GAD), strong staining indicated inhibitory axon transmission (Brewer et al., 2013). The role of EC-CA1 is evaluated as feed-forward because it is most likely an excitatory pathway (Steward, 1976). The spatiotemporal organization of spikes may provide insight to the function and behavior of the hippocampal network. As previously found in Vakilna et al. (2021), spontaneous interspike intervals (ISI) followed different log-log distributions between subregions and in the axons between regions (**Fig 2**). A linear model was fit to the data by Eq.2 to describe the probability (P) of a spike firing at time (t) after a previous spike and m as the slope of the fit. Thus, graphs with steeper slopes represent shorter times which represent faster firing rates. **Fig 2** shows the spike timing in the four-tunnel architecture compared to the five-tunnel architecture as cumulative probability distributions of inter-spike intervals (ISI), both in feed-forward and feedback axons by subregion. All distributions were well-fit by a single exponential slope with $R^2>0.99$, suggesting a common mechanism of spike timing. All slopes were significantly different by ANCOVA followed by Tukey-HSD. **Fig 2F** shows that most of the four-tunnel feed-forward slopes were significantly different, as were most of the five-tunnel slopes (**Fig 2G**). **Fig 2H** compares the slopes of the 4 to the 5 tunnel architectures where spike rates were 17% faster for the four-tunnel vs. the five-tunnel configuration at the first EC-DG stage of transmission. In all other connections, slopes in the five-tunnel design were 30-87 % faster than the four-tunnel axon architectures. This means that spike rates were higher overall in the five-tunnel compared to the four-tunnel configuration.

Fig 2. Axonal cumulative probability distributions of inter-spike intervals (ISI) and slopes in the four-tunnel architecture.

A-H) feed-forward (FF) **A)** EC-DG four-tunnel has 17% faster spiking than five tunnel FF ISI slope. **B)** DG-CA3 five tunnel has 42% faster spiking than four-tunnel FF ISI slope. **C)** CA3-CA1 five-tunnel has 78% faster spiking than four-tunnel FF ISI slope. **D)** CA1-EC five tunnel has 87% faster spiking than four-tunnel FF ISI slope. **E)** EC-CA1 five tunnel has 30% faster spiking than four-tunnel FF ISI slope. **F)** All four-tunnel feed-forward slopes are significantly different. **G)** Most of the five tunnel slopes are significantly different. **H)** All slopes are significantly different between four-tunnel and five tunnel. **I-N)** feedback (FB) **I)** DG-EC five tunnel has 16% faster spiking than four-tunnel FB ISI slope. **J)** CA3-DG four-tunnel has 12% faster spiking than five tunnel FB ISI slope. **K)** CA1-CA3 four-tunnel has 1% faster spiking than five tunnel FB ISI slope. **L)** Most of the four-tunnel feedback slopes are significantly different except CA3-DG and CA1-CA3. **M)** Five tunnel CA1-CA3 slope is significantly different from other tunnels. **N)** All slopes are significantly different between four and five tunnel. P-values calculated using ANCOVA followed by Tukey-HSD. Numbers within bars represent total ISIs from 9 and 6 arrays, respectively.

Fig 2I-N shows the spike timing in feedback axons. In **Fig 2L,** both CA3 feedback into DG and CA1 into CA3 spike rates were higher than DG to EC feedback in the four-tunnel architecture. Feed-back spike rates within five-tunnel axons were increased by 16% for DG back to EC compared to the other feedback routes (**Fig 2M**). In **Fig 2N**, we compare feedback rates for 4 vs. five-tunnel architectures where spike rates differed by only -12 to +16%, much less than the -17 to 87% differences in the feed-forward configuration (**Fig 2H**). **Supplementary Fig 2** shows the same data rearranged to compare different inter-subregion tunnel axons.

Supplementary Fig 2. Axonal cumulative probability distributions of inter-spike intervals (ISI) and slopes in the four-tunnel (*n* = 9 arrays) and five tunnel architecture (*n* = 6 arrays) compared by subregions.

A-B) feed-forward (FF) **C-D)** feedback (FB) **E-F)** subregional.

We conducted similar analyses of the subregions that either send or receive the axons monitored in the tunnels (**Fig 3**). For four-tunnel networks, comparison of **Fig 3A-D** shows the slowest spiking in CA3 (**Fig 3E**). Similar comparisons for the five-tunnel networks show the fastest spiking in EC compared to the other subregions (**Fig 3F**). Comparison of the two tunnel types in **Fig 3G**, shows only a 4% higher spike rate in EC for the five-connect networks, while all the others produced an 11-80% advantage for the four-connect networks over those of the five-connect. Next, we explored the difference in grouping of spikes into bursts and its impact on network communication.

Fig 3. Subregional cumulative probability distributions of inter-spike interval (ISI) slopes in the four-tunnel (*n* = 9 arrays) and five tunnel architecture (*n* = 6 arrays).

The four-tunnel axon architecture promotes faster subregional spiking than the five tunnel axon architectures in all subregions. **A-G)** subregional **A)** EC five-tunnel has 57% slower spiking than four-tunnel ISI slope. **B)** DG five-tunnel has 37% slower spiking than four tunnel ISI slope. **C)** CA3 five-tunnel has 45% slower spiking than four tunnel ISI slope. **D)** CA1 five-tunnel has 54% slower spiking than four tunnel ISI slope. **E)** Most four-tunnel subregion slopes were significantly different from each other except EC and CA1. **F)** Five tunnel subregion slopes were similar except for differences from DG, and DG vs CA1. **G)** All subregions are significantly

different between four-tunnel and five tunnel. P-values calculated using ANCOVA followed by Tukey-HSD. Numbers within bars represent total ISIs from 9 and 6 arrays, respectively.

Faster bursting overall in four-tunnel versus five-tunnel architecture supports greater information transfer in the four-tunnel axon architecture

Spikes are organized into bursts or packets of information for both local and global computation and routing (Graham and Rockmore, 2011; Graham, 2014; Luczak et al., 2015). These bursts of spikes are easily recorded in vivo because of their intrinsic function in raising somal calcium concentration (Jimbo et al., 1993). Each sub-region of the hippocampus is responsible for a different set of computations that give rise to learning and memory (Kesner and Rolls, 2015). The spatiotemporal bursting dynamics in sub-regions and axons is key for insight into each sub-region's function in routing information for processing. Previously, we found spontaneous interburst intervals (IBI) followed a two-part log–log distribution suggesting faster up and slower down states that vary sub-regionally and between regions (Vakilna et al., 2021). Like ISI, a decrease in slope indicates a slower frequency of bursting.

Feed-forward fast bursting (**Fig 4 & 5**) and slow bursting generally decreased in five-tunnel compared to four-tunnel axon architecture. Bursts were plotted over a timescale of two orders of magnitude representing probability vs. times at which a bursting event occurred. Here, we focused on fast bursts as they represent most events. In the four-tunnel axons, bursting was slowest in the EC-DG (**Fig 4A**) and CA3-CA axons (**Fig 4C**), as compared in **Fig 4E**. Among five-tunnel axons, these regions were joined in slower bursting by EC-CA1 and EC-CA3 (**Fig 4G**). In comparison to the four-tunnel architecture (**Fig 4H**), feed forward five-tunnel networks burst rates were 30-76% slower with EC-CA1 showing the largest difference. **Figs 4I, J, K** compare the slopes of the

feedback bursting. In the four-tunnel networks (**Fig 4L**), feedback bursting was lowest in the DG to EC networks were 52-56% smaller than in the four-tunnel networks (**Fig 4L, M**).

Fig 4. Smaller differences in interburst interval (IBI) slopes in inter-regional axon distributions in the four-tunnel (*n* = 9 arrays) and five tunnel architecture (*n* = 6 arrays). The five tunnel feed-forward axon architecture shows a switch from short interburst intervals to long interburst intervals earlier than the four-tunnel axon architectures. **A-H)** feed-forward (FF) **A)** EC-DG fast fit four-tunnel has 30% faster bursting than five tunnel FF IBI slope. **B)** DG-CA3 fast fit four-tunnel has 38% faster bursting than five tunnel FF IBI slope. **C)** CA3-CA1 fast fit had no significant difference between four-tunnel and five tunnel architectures. **D)** CA1-EC fast fit four-tunnel has 36% faster bursting than five tunnel FF IBI slope. **E)** EC-CA1 fast fit four-tunnel has 76% faster bursting than five tunnel FF IBI slope. **F)** Four-tunnel feed-forward slopes are significantly different except DG-CA3 and EC-CA1. **G)** Most of the five tunnel slopes are significantly different. **H)** All slopes are significantly different between four-tunnel and five-tunnel except CA3-CA1. **I-N)** feedback (FB) I**)** DG-EC fast fit four-tunnel has 52% faster bursting than five tunnel FB IBI slope. **J)** CA3-DG fast fit four-tunnel has 52% faster bursting than five tunnel FB IBI slope. **K)** CA1-CA3 fast fit four-tunnel has 56% faster bursting than five tunnel FB IBI slope. **L)** All four-tunnel feedback slopes are significantly different. **M)** Five-tunnel feedback slopes are significantly different except CA1-CA3 and CA3-EC. **N)** All slopes are significantly different between four and five tunnel. P-values calculated using ANCOVA followed by Tukey-HSD. POI (position of intersect) is the intersection of the fast fit and slow fits. Numbers within bars represent total IBIs from 9 and 6 arrays, respectively.

Sub-regions showed an overall decrease in bursting in the five-tunnel compared to the four-tunnel networks (**Figs 5A, B, D, E**). Subregions EC, DG and CA1 neurons burst at 80-100% higher rates than CA3 in the high-rate segments of four-tunnel architectures and burst at 42-76% slower rates (longer interburst intervals) in the five-tunnel architecture (**Fig 5F, G**) with CA1 showing the largest difference compared to their four-tunnel counterparts.

Fig 5. Subregional interburst interval (IBI) distributions of slopes in the five-tunnel architecture (*n* = 6 arrays) burst faster than four-tunnel architecture (*n* = 9 arrays). **A-G)** subregional **A)** EC fast fit five-tunnel has 70% slower bursting than four-tunnel IBI slope. **B)** DG fast fit five-tunnel has 66% slower bursting than four-tunnel IBI slope. **C)** CA3 fast fit five-tunnel has 40% slower bursting than four-tunnel IBI slope. **D)** CA1 fast fit five-tunnel has 66% slower bursting than four-tunnel IBI slope. **E)** All four-tunnel subregion slopes were significantly different from each other except DG vs CA1. **F)** All five-tunnel subregion slopes were significantly different from each other except EC vs DG and DG vs CA1. **G)** All subregions are significantly different between four-tunnel and five-tunnel. P-values calculated using ANCOVA followed by Tukey-HSD. POI is the intersection of the fast fit and slow fits. Numbers within bars represent total IBIs from 9 and 6 arrays, respectively.

CA3-CA1 and CA1-EC Feed-Forward Burstiness Increases in the Presence of the EC-CA3 Perforant Pathway

We measured the burstiness in axons and soma in our networks as the fraction of spikes in bursts for each subregion and interregion, a distribution of burstiness in axons and soma were gathered and the fraction of units with spikes in bursts above 50% was calculated.

Under the paradigm of the packet switching brain, bursts represent parts of an encoded message. This can be interpreted as active processing (Luczak et al 2015; Graham 2014). With the addition of the five-tunnel architecture, we observed an increase in feed-forward burstiness in the EC-CA3 feed forward and CA1-EC feed forward pathways by comparison through a nonparametric Kruskal-Wallis, a test for differences between two distributions. (**Fig. 6A**). *In vivo*, the perforant pathway from the EC to CA3 is responsible for encoding ques that can reconstruct memories, those memories are then organized in the CA1 and back propagated to the EC and to the cortex for higher level processing. With the addition of the EC-CA3 pathway, we can now see encoded messages being sent from the CA3 to the CA1 and the CA1 sending those messages to the EC. There were no changes in burstiness in the feedback pathways (**Fig. 6B**). The burstiness of the DG and CA1 subregions decreased in the five-tunnel design (**Fig. 6C**).

Fig. 6 Percentage of axons with spikes appearing in bursts (frBurst) comparing 4 vs. 5 tunnel arrays were higher in CA3-CA1 and CA1-EC feed forward tunnels.

Counts were normalized by the number of arrays for each design. A Kruskal Wallis test was performed to assess significant changes in burstiness. A) Burstiness in four-tunnel and five-tunnel architecture for feed-forward axons. The CA3-CA1 feed forward tunnels and the CA1-EC feed forward tunnels had significantly higher Fractional Spikes In Bursts (frBurst) in the five-tunnel arrays than the four-tunnel arrays. B) Burstiness for 4v5 arrays for feedback axons. There are no significant differences between the two array designs. C) Burstiness for subregional spikes. Five-tunnel arrays had fewer active units. CA3 and CA1 had significantly lower burstiness according to ANOVA. DG had significance on the cusp for lower burstiness in the five-tunnel architecture.

Discussion

More inter-regional feed-forward spiking but less inter-regional bursting with feed-forward EC-CA3 inputs versus four-tunnel architecture without CA3 input

Using a novel four-compartment device, we were able to reconstruct a hippocampal network comprised of the entorhinal cortex, dentate gyrus, CA3, and CA1 subregions in a loop with axonal connections isolated in microfluidic tunnels. Each tunnel and subregion were equipped with electrodes that enabled the monitoring of the direction of axonal propagation. Previously, connectivity of the trisynaptic circuit was inferred through changes in somal activity of other sub-regions. This novel system used self-wiring that allowed differences in axonal spike dynamics to be measured by sub-region. All spiking dynamics followed linear log-log distributions, displaying the canonical non-Gaussian distributions inherent to computations in neural systems (Beggs and Timme, 2012). As previously reported by Vakilna et al. (2021), interburst intervals required two slopes to fit a linear log-log model, suggesting up- and down-states of network computation.

Between the four-tunnel and five-tunnel model, the five-tunnel architecture showed an increase in feed-forward firing, but bursting decreased by up to 76% in both soma and axons in the feed-forward and feedback directions. This suggests tighter control of spiking and more precise communication between subregions. Information transfer in the five-tunnel appears more organized than in the four-tunnel network, as we have seen with these same networks after stimulation (Lassers et al., 2023).

Function of the EC-CA3 perforant path

The EC-CA3 connection in the perforant pathway plays a crucial role in modulating

hippocampal dynamics and functionality. Activation of the CA3 region is largely dependent on feed-forward excitatory inputs from direct perforant path fibers (Biagini et al., 2008). Our networks show a large amount of feed-forward activity between all subregions, following the canonical representation of the trisynaptic loop. The five-tunnel architecture showed 30-87% greater interregional feed-forward spiking with the CA1-EC connection showing the greatest increase. Lesions in the CA3 region have been shown to impair spatial recall without significantly modifying CA1 activity, while lesions in the EC input led to mild impairments despite reducing CA1 spatial selectivity (O'Reilly 2014). Therefore, the EC-CA3 seems to enable spatial navigation. Feedback spike rates differed to a much smaller extent from 12 to 16% compared to the feed-forward configuration. Compared by subregions, network differences produced an 11-80% advantage in axonal spiking for the four-tunnel architecture over the five-tunnel architecture. Degradation of these fibers is correlated with increased CA3 hyperactivity (Cappaert et al., 2015). It may be that inputs from the EC help regulate CA3 in a more precise fashion. This is supported by the 11% gain in feed-forward activity of the CA3 subregion in the five-tunnel configuration.

Balancing and modulating excitatory/inhibitory (E/I) activity is crucial for efficient memory encoding (Zhou and Yu, 2018). Spontaneous firing patterns of neurons help understand network states and stimulus representation. Achieving global E/I balance requires sparsely connected neurons with strong inhibitory connections to prevent excessive excitation (Zhou and Yu, 2018). Pyramidal neurons maintain optimal firing rates for information processing, while inhibitory interneurons respond over a wider range, aiding in local homeostasis and sub-regional coordination range (Maffei and Fontanini, 2009). The minimal differences we found in feedback spiking dynamics suggest that excitatory and inhibitory signals are more balanced in the five-tunnel architecture.

In contrast, the five-tunnel architecture produced burst rates 30-76% slower than the four-tunnel architecture, both interregional and by subregion, with CA1 and CA1 connections showing the greatest difference in burst rates. The brain's circuits do not produce the same response to identical input each time. This variability reflects brain network plasticity. Information in the brain, especially between hippocampal sub-regions, is likely transmitted in bursts of action potentials. These bursts can be compared to packets in Internet communication, where information is sent in parts and then reassembled or interpreted at a specific location, enhancing message reliability (Graham and Rockmore, 2011). In cortical circuits, bursting packets are essential for information transmission. The number and timing of spikes within these bursts convey details about stimulus types, as was previously identified between the DG and CA3 (Bhattacharya et al., 2016). Control experiments by Brewer et al. (2013) revealed unique spike dynamics between DG-CA3, distinct from DG-DG and CA3-CA3, indicating that specific anatomical structures result in distinct coding patterns.

The addition of the EC-CA3 connection decreased bursting IBI in the five-tunnel architecture and specifically in CA1 axons. Burst activation for the EC-CA1 pathway is known to enhance long-term potentiation (O'Reilly et al., 2014). However, increased spiking in this pathway suggests spike timing may be better controlled with more spikes outside bursts. Despite minor changes in spike dynamics in DG-EC feedback, the higher number of spikes may indicate the development of more inhibitory neurons in DG to more finely control network dynamics. The overall differences in spiking and bursting rates between four and five-tunnel architectures suggest there may be a balancing mechanism related to the spatial-temporal organization of spikes.

The packet paradigm of information transfer in networks

Based on bursting spikes associated with behavior in the rat brain, bursts are proposed to operate as packets of information transfer (Luczak et al. 2015). The theoretical framework of Graham and Rockmore (2011) proposes that certain brain networks have the ability to "packet switch" analogous to packets of data transferred over the Internet. Packets are pieces of a whole message where each piece is sent along the most efficient route and recombined at the target. When a packet is received, the target sends a message back confirming its receipt. This reduces the delay of the message being received and increases the bandwidth of the communication network. In this metaphor, packets are analogous to bursting activity in biological neural networks and feedback network activity could function like a receipt of information. The most interesting difference we have seen in comparing our addition of an EC-CA3 connection in the network design is the increase in burstiness of the CA3-CA1 feed-forward connection with more spikes and bursts per array than in the four-tunnel design. This suggests that the EC-CA3 connection enhances the sharpness or strength of information routing from the CA3 to the CA1 by tightening the specificity of spiking within bursts.

Neuromorphic computing applications

Neuromorphic computing is a bio-inspired computing paradigm that leverages event-based computing and neuronal architectures to solve brain-like problems including deep learning and continuous learning at low power consumption. Recent advances in artificial neural network architectures have started to explore layer skipping as a way to avoid optimization problems and generalize the performance of neural networks with less information loss (Oyedontun et al., 2023). The information skipped past the previous layer is re-integrated after the hidden layer operations. However, the reasons why layer skipping creates the ability to train very deep networks are unclear.

Layer skipping is intrinsic to the hippocampus through the EC-CA3 pathway studied here and may act as a way to prime downstream neurons for input from the DG to the CA3. Our laboratory is beginning investigations into hippocampus-inspired neural networks as a way to create generalizable auto-associative *in silico* neural networks. These results suggest that more collaboration between the neuroscience and the machine learning communities for new computing architectures could accelerate advances in artificial intelligence.

Limitations

The *in vitro* model network developed to study spike dynamics may not accurately reflect the complexity of *in vivo* conditions due to several reasons. The culture is two-dimensional, simplifying the complex 3D axonal organization seen *in vivo*, which enables more sophisticated signal integration. Although some functional connectivity in the hippocampus occurs within a specific thickness, the *in vitro* model lacks the laminated arrangement of neurons seen in the hippocampus, affecting axonal innervation patterns in specific dendritic layers. 3D cultures, which more closely resemble *in vivo* systems, show greater response to stimuli but have not yet achieved isolated axon cultures as with the current model. The model also faces limitations in electrophysiological monitoring and the number of neurons and axon tunnels that can be analyzed at once, affecting the thoroughness of functional connection analysis. Anatomical differences from *in vivo* systems include the lack of inputs from other brain regions, including modulatory cholinergic and dopaminergic inputs. Future work includes improving the model's 3D culture and electrophysiological monitoring capabilities and using optogenetics for defined multicell patterned stimulation to more closely mimic *in vivo* conditions.

Conclusion

Compared to the four-tunnels, the five-tunnel architecture with perforant path-like EC-CA3 connections had shorter inter-spike interval distributions balanced by longer interburst intervals. Differences in spiking in each of the four subregions and in their communicating, axons suggested functional differences in information processing between subregions and between the four-tunnel and five-tunnel architecture. The spike dynamics from addition of the EC-CA3 pathway suggested more precise communication between subregions. This was further supported by the burstiness of CA3-CA1 and CA1-EC pathways increasing with the five-tunnel design, suggesting that information packet coding was increased.

Data Availability Statement

The code and data are available on GitHub.

Ethics Statement

The animal study was approved by UC Irvine Institutional Animal Care and Use Committee. The study was conducted in accordance with the local legislation and institutional requirements.

Author contributions

GB and SL designed the study. RC fabricated the devices. GB and RC isolated and cultured the neurons. RC and YV collected spike data. SK and SL analyzed the data with direction from GB and WT. SK wrote the initial draft of the manuscript, which was revised by GB, SL, YV and WT. All authors assisted with reviewing and approval of the final manuscript.

Conflict of Interest

The authors declare that the research was conducted in the absence of any commercial or financial relationships that could be construed as a potential conflict of interest.

Funding

The author(s) declare financial support was received for the research, authorship, and/or publication of this article. This study was supported in part by funds from the UC Irvine Foundation. The Foundation had no decision making capacity for the design or manuscript.

References

Andersen P, Bliss TV, Skrede KK. Lamellar organization of hippocampal pathways. Exp Brain Res. 1971;13(2):222-38.

Beggs JM, Timme N. Being critical of criticality in the brain. Front Physiol. 2012;3:163.

Bhattacharya A, Desai H, DeMarse TB, Wheeler BC, Brewer GJ. Repeating Spatial-Temporal Motifs of CA3 Activity Dependent on Engineered Inputs from Dentate Gyrus Neurons in Live Hippocampal Networks. Front Neural Circuits. 2016;10:45.

Biagini G, Baldelli E, Longo D, Contri MB, Guerrini U, Sironi L, et al. Proepileptic influence of a focal vascular lesion affecting entorhinal cortex-CA3 connections after status epilepticus. J Neuropathol Exp Neurol. 2008;67(7):687-701.

Boehler MD, Wheeler BC, Brewer GJ. Added astroglia promote greater synapse density and higher activity in neuronal networks. Neuron Glia Biol. 2007;3(2):127-40.

Braitenberg V. ANATOMICAL BASIS FOR DIVERGENCE, CONVERGENCE, AND INTEGRATION

IN THE CEREBRAL CORTEX. In: Grastyán E, Molnár P, editors. Sensory Functions: Pergamon; 1981. p. 411-9.

Brewer GJ, Boehler MD, Jones TT, Wheeler BC. NbActiv4 medium improvement to Neurobasal/B27 increases neuron synapse densities and network spike rates on multielectrode arrays. J Neurosci Methods. 2008;170(2):181-7.

Brewer GJ, Boehler MD, Leondopulos S, Pan L, Alagapan S, DeMarse TB, et al. Toward a self-wired active reconstruction of the hippocampal trisynaptic loop: DG-CA3. Front Neural Circuits. 2013;7:165.

Brewer GJ, Boehler MD, Pearson RA, DeMaris AA, Ide AN, Wheeler BC. Neuron network activity scales exponentially with synapse density. J Neural Eng. 2009;6(1):014001.

Brewer GJ, Cotman CW. Survival and growth of hippocampal neurons in defined medium at low density: advantages of a sandwich culture technique or low oxygen. Brain Res. 1989;494(1):65-74.

Cappaert NLM, Van Strien NM, Witter MP. Chapter 20 - Hippocampal Formation. In: Paxinos G, editor. The Rat Nervous System (Fourth Edition). San Diego: Academic Press; 2015. p. 511-73.

Chaure FJ, Rey HG, Quian Quiroga R. A novel and fully automatic spike-sorting implementation with variable number of features. J Neurophysiol. 2018;120(4):1859-71.

Chen R, Vakilna YS, Lassers SB, Tang WC, Brewer G. Hippocampal network axons respond to patterned theta burst stimulation with lower activity of initially higher spike train similarity from EC to DG and later similarity of axons from CA1 to EC. J Neural Eng. 2023;20(5).

de Camargo RY, Recio RS, Reyes MB. Heteroassociative storage of hippocampal pattern sequences in the CA3 subregion. PeerJ. 2018;6:e4203.

Graham D, Rockmore D. The packet switching brain. J Cogn Neurosci. 2011;23(2):267-76.

Graham DJ. Routing in the brain. Front Comput Neurosci. 2014;8:44.

Jimbo Y, Robinson HP, Kawana A. Simultaneous measurement of intracellular calcium and electrical activity from patterned neural networks in culture. IEEE Trans Biomed Eng. 1993;40(8):804-10.

Kesner RP, Rolls ET. A computational theory of hippocampal function, and tests of the theory: New developments. Neurosci Biobehav R. 2015;48:92-147.

Lassers SB, Vakilna YS, Tang WC, Brewer GJ. The flow of axonal information among hippocampal sub-regions 2: patterned stimulation sharpens routing of information transmission. Front Neural Circuit. 2023;17.

Luczak A, McNaughton BL, Harris KD. Packet-based communication in the cortex. Nat Rev Neurosci. 2015;16(12):745-55.

Maffei A, Fontanini A. Network homeostasis: a matter of coordination. Curr Opin Neurobiol. 2009;19(2):168-73.

Narula U, Ruiz A, McQuaide M, DeMarse TB, Wheeler BC, Brewer GJ. Narrow microtunnel technology for the isolation and precise identification of axonal communication among distinct hippocampal subregion networks. PLoS One. 2017;12(5):e0176868.

O'Reilly KC, Alarcon JM, Ferbinteanu J. Relative contributions of CA3 and medial entorhinal cortex to memory in rats. Front Behav Neurosci. 2014;8:292.

Oyedotun OK, Ismaeil KA, Aouada D. Why Is Everyone Training Very Deep Neural Network With Skip Connections? IEEE Trans Neural Netw Learn Syst. 2023;34(9):5961-75.

Poli D, Thiagarajan S, DeMarse TB, Wheeler BC, Brewer GJ. Sparse and Specific Coding during Information Transmission between Co-cultured Dentate Gyrus and CA3 Hippocampal

Networks. Front Neural Circuits. 2017;11:13.

Poli D, Wheeler BC, DeMarse TB, Brewer GJ. Pattern separation and completion of distinct axonal inputs transmitted via micro-tunnels between co-cultured hippocampal dentate, CA3, CA1 and entorhinal cortex networks. J Neural Eng. 2018;15(4):046009.

Newman MEJ. Power laws, Pareto distributions and Zipf's law. Contemporary Physics. 2005;46(5):323-51.

Steward O. Topographic organization of the projections from the entorhinal area to the hippocampal formation of the rat. J Comp Neurol. 1976;167(3):285-314.

Vakilna YS, Tang WC, Wheeler BC, Brewer GJ. The Flow of Axonal Information Among Hippocampal Subregions: 1. Feed-Forward and Feedback Network Spatial Dynamics Underpinning Emergent Information Processing. Front Neural Circuit. 2021;15.

Zhou S, Yu Y. Synaptic E-I Balance Underlies Efficient Neural Coding. Front Neurosci. 2018;12:46.

Figure 1

Click here to access/download;Figure;Fig1.TIF

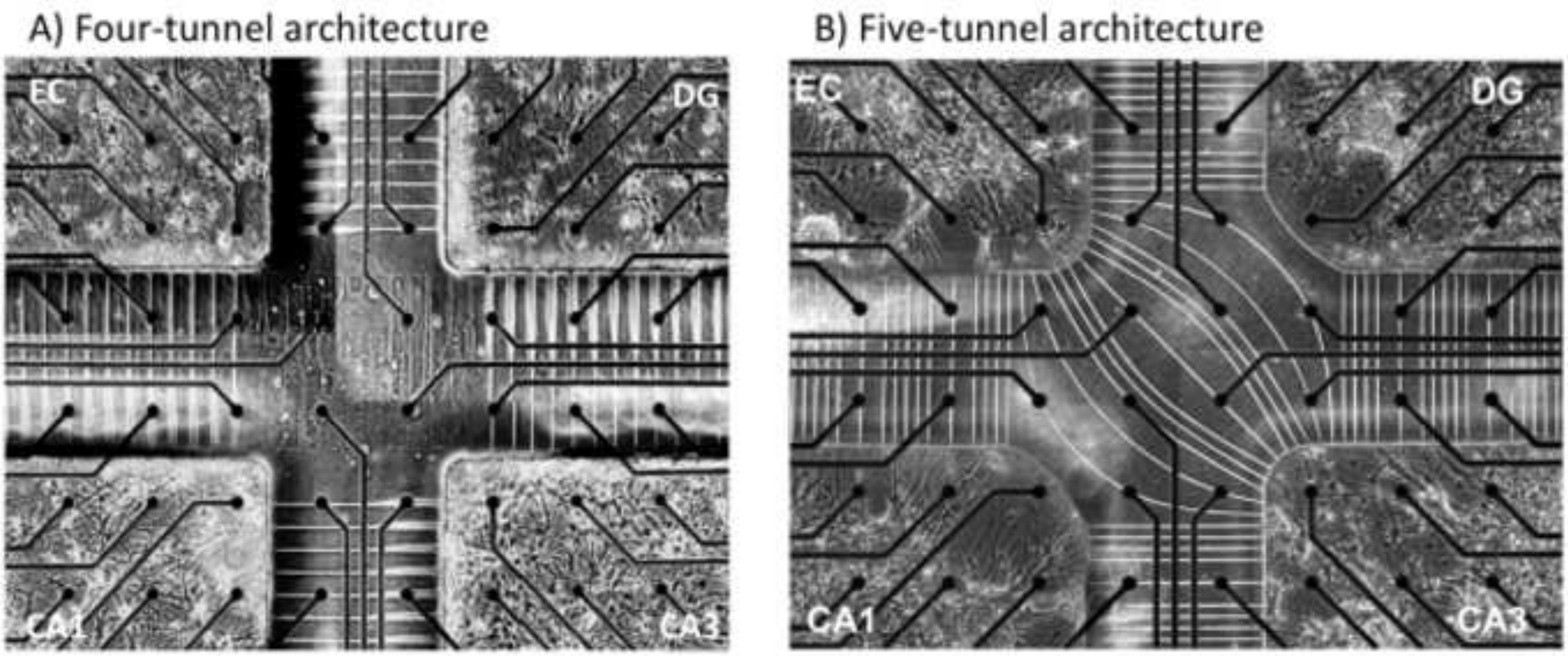

Figure 2

Click here to access/download;Figure;Fig2.TIF

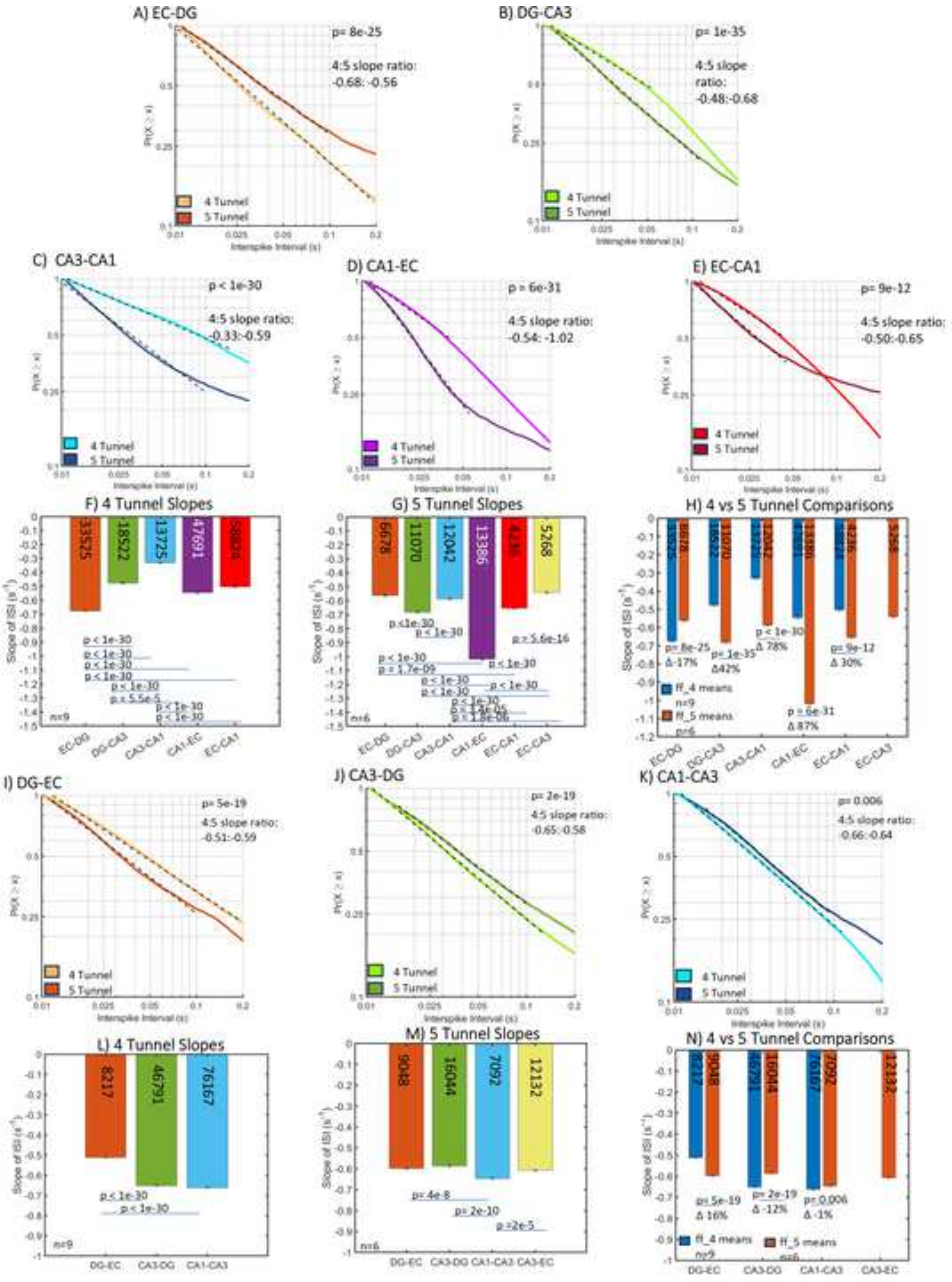

Figure 3 Click here to access/download;Figure;Fig3.TIF

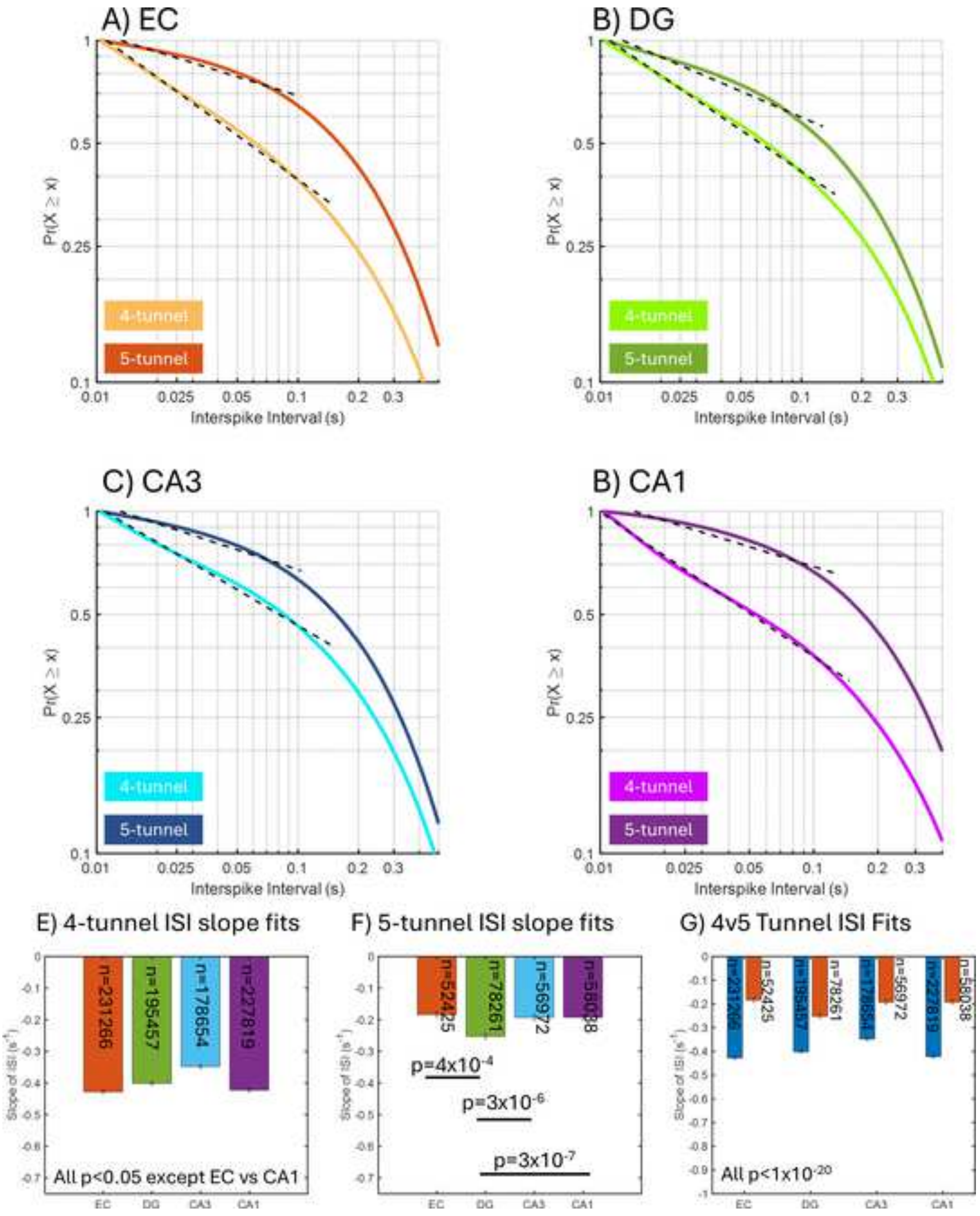

Figure 4

Click here to access/download;Figure;Fig4.TIF

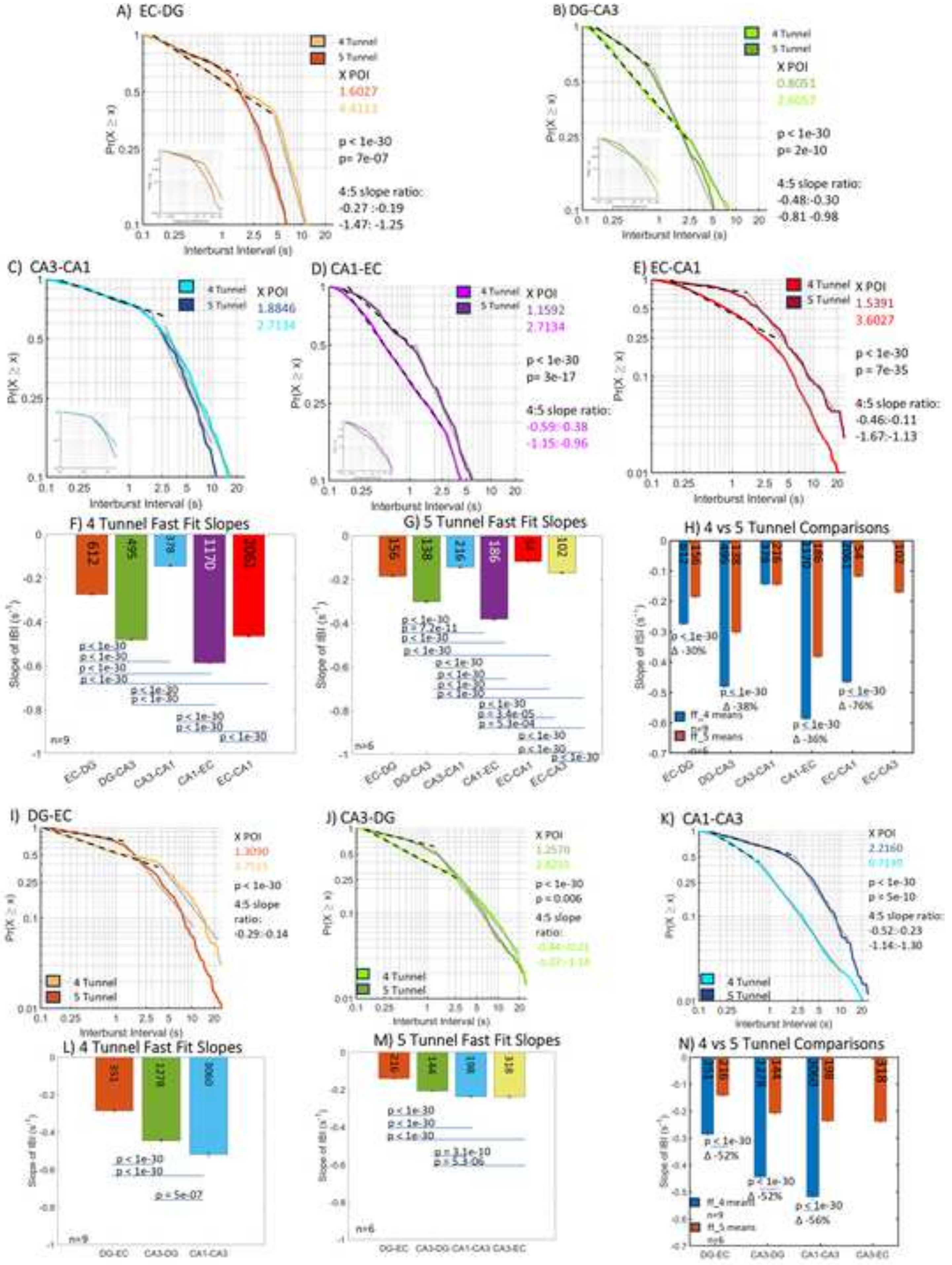

Click here to access/download;Figure;Fig5.TIF

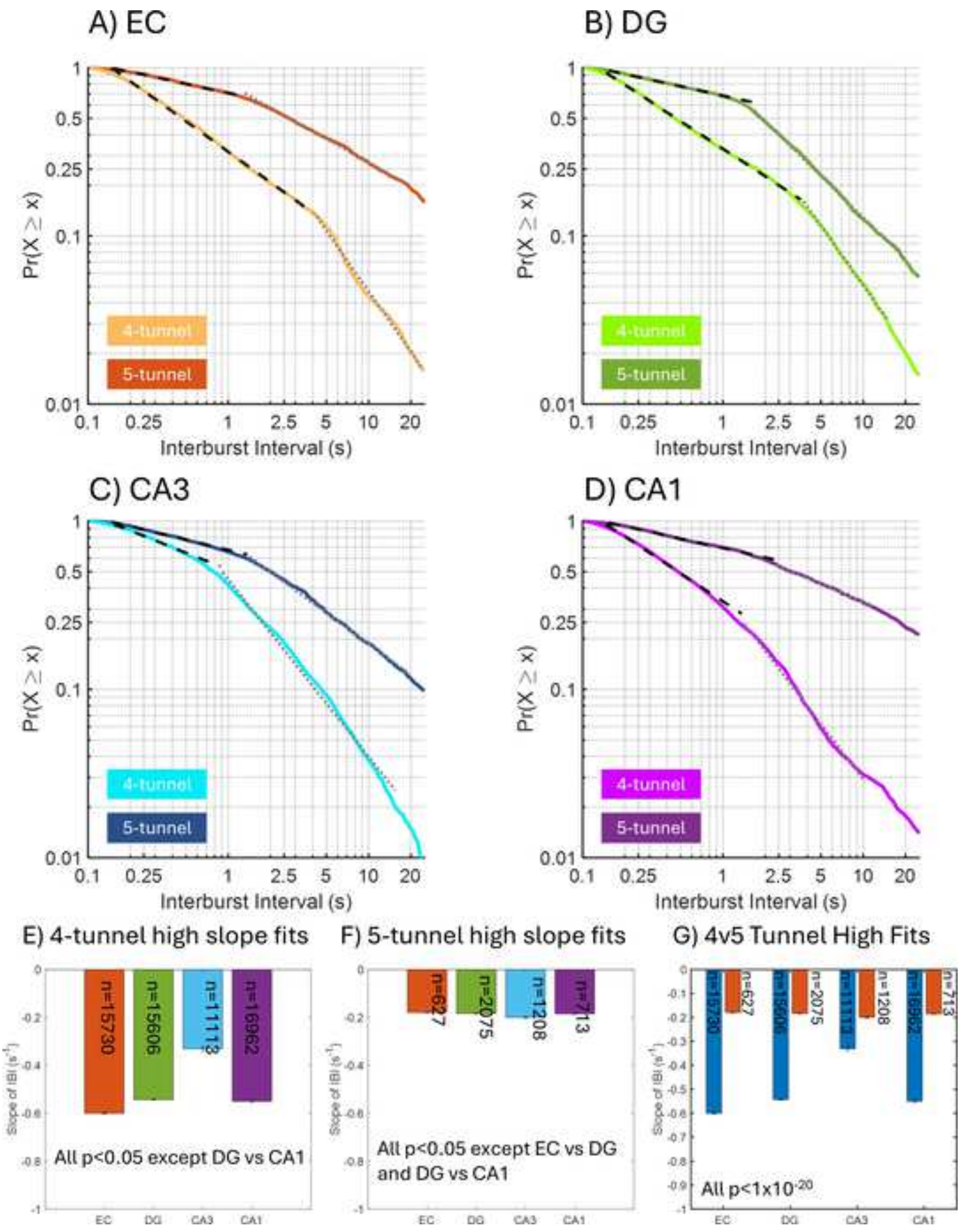

Figure 6

Click here to access/download;Figure;Fig6.TIF

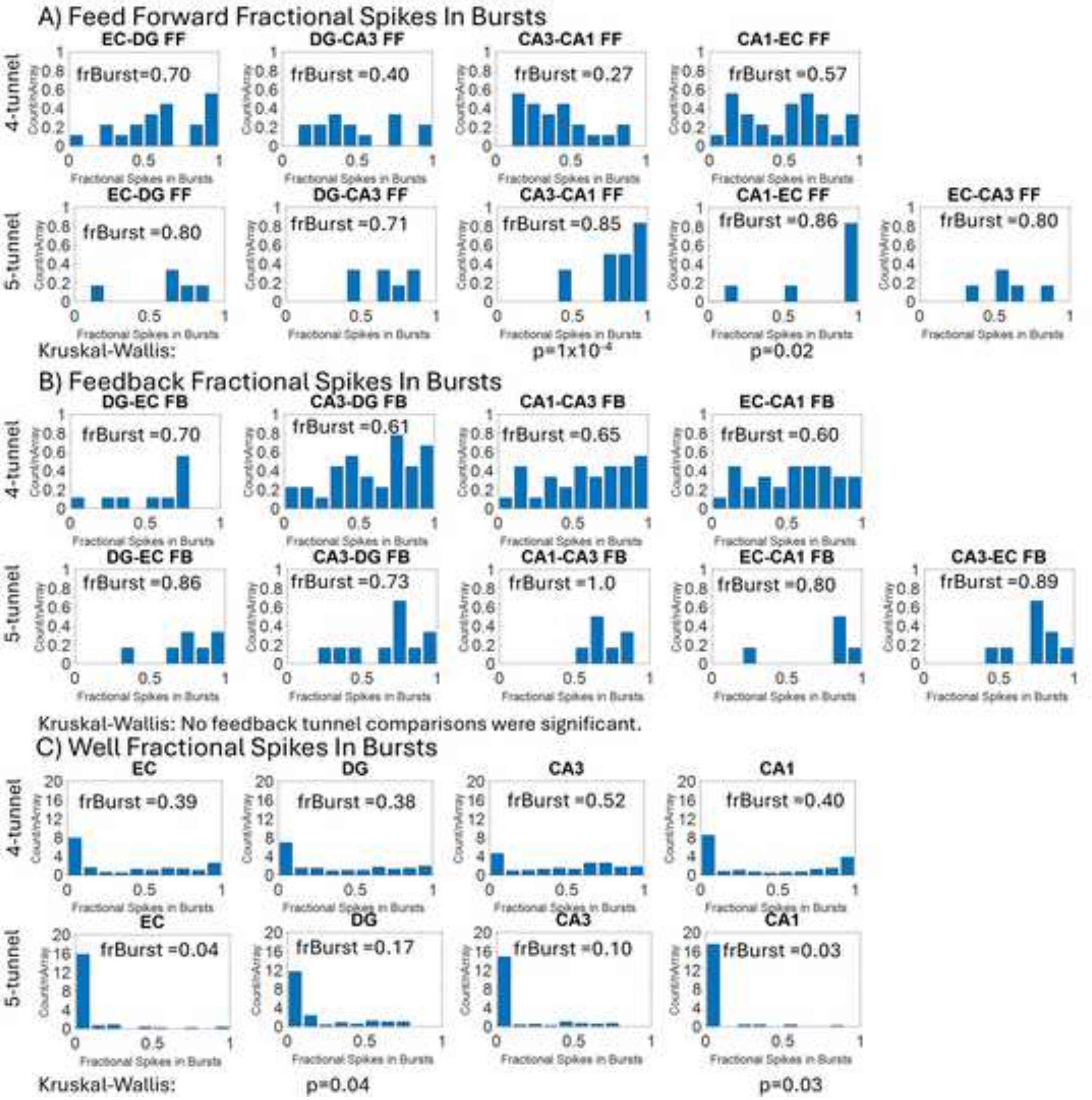

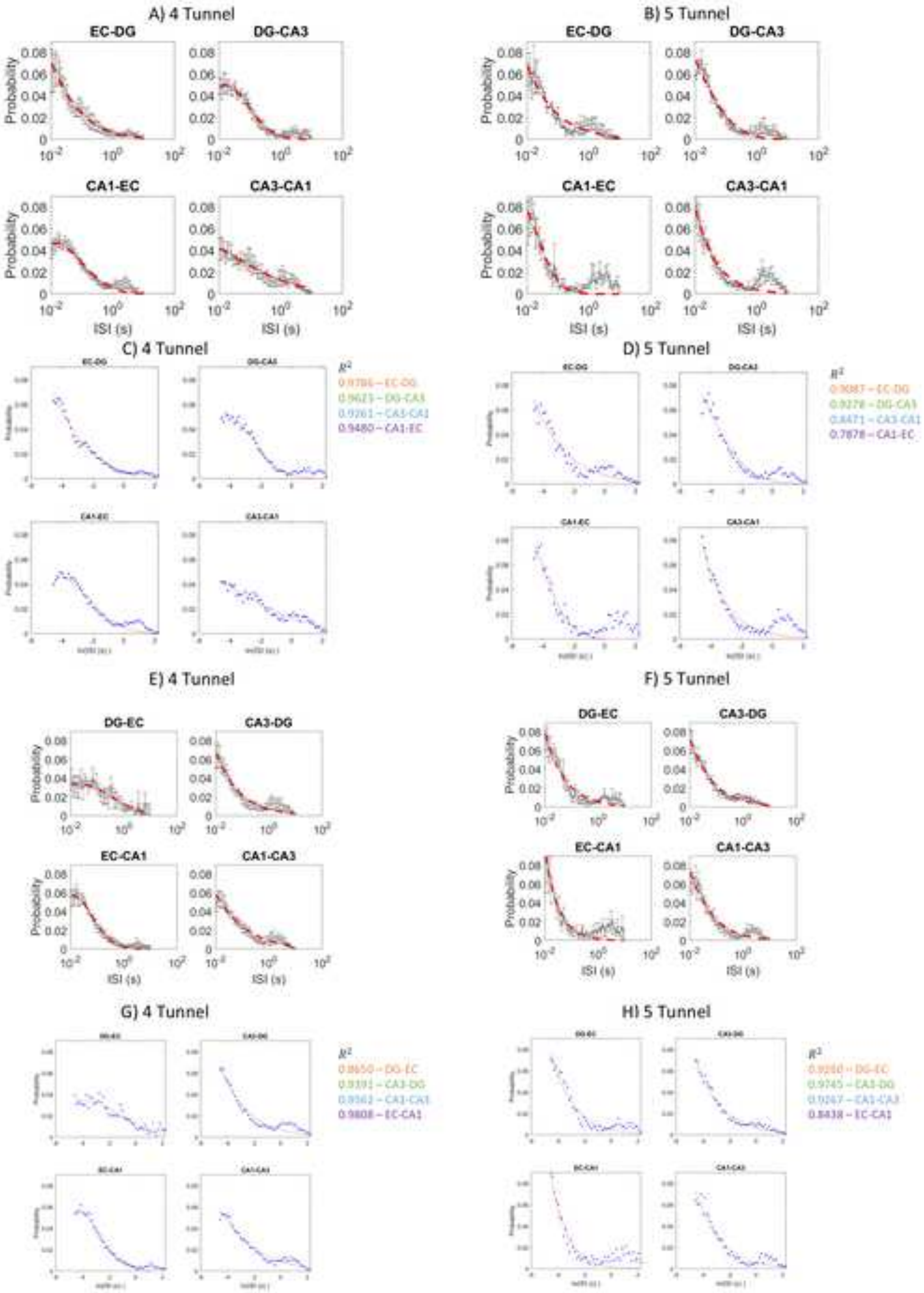
A) 4 Tunnel
B) 5 Tunnel
EC-DG
DG-CA3
CA1-EC
CA3-CA1
Probability
ISI (s)
C) 4 Tunnel
D) 5 Tunnel
R^2
E) 4 Tunnel
F) 5 Tunnel
DG-EC
CA3-DG
EC-CA1
CA1-CA3
G) 4 Tunnel
H) 5 Tunnel

Click here to access/download;Figure;SupFig2.TIF

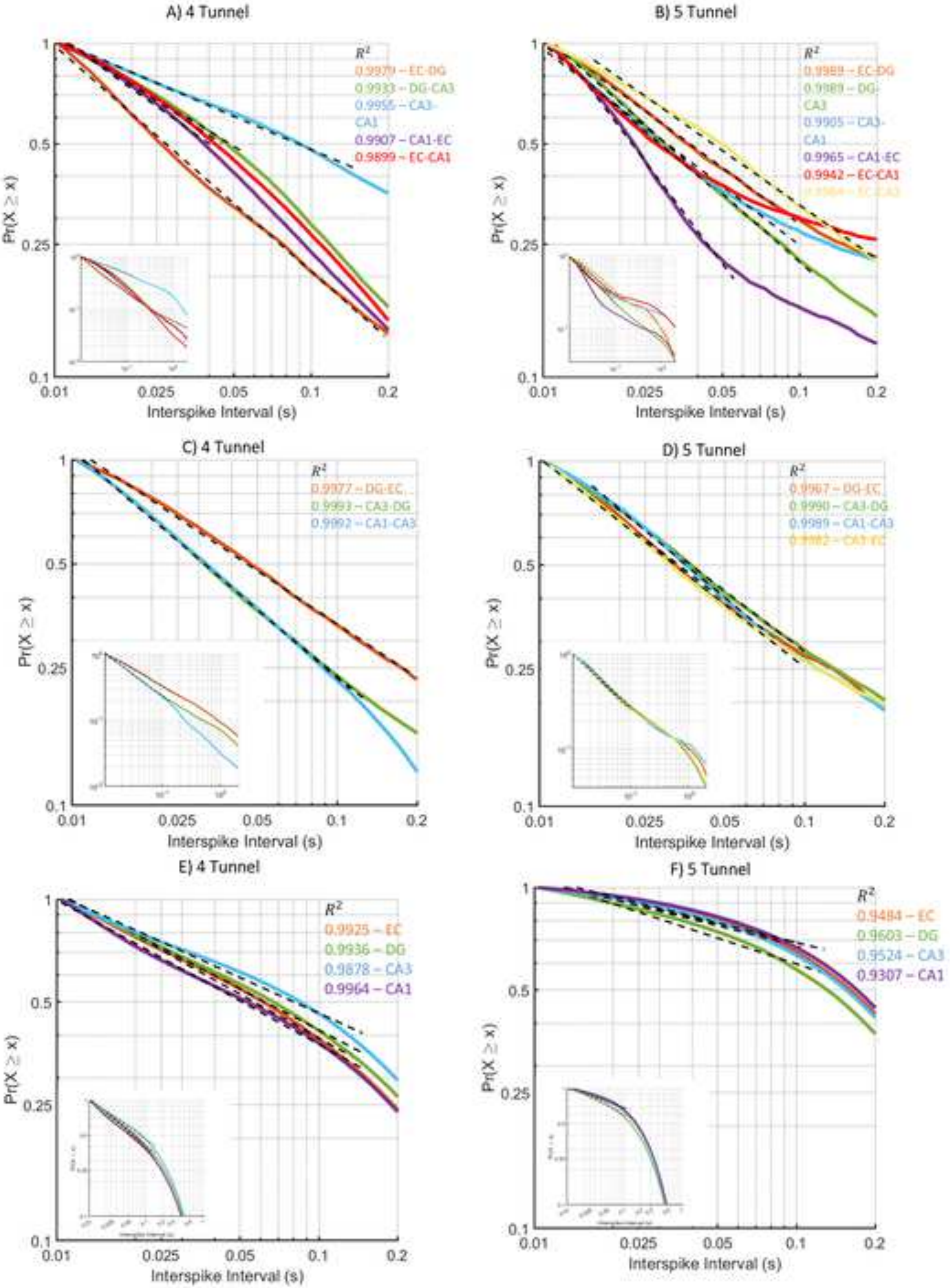

Data Review URL

Click here to download Data Review URL http://github.com/Brewer-Neurolab/Khatri_Lassers_EC_CA3_Pathway_Effect
