# Supplementary material for: Impact of the entorhinal feed-forward connection to the CA3 on hippocampal coding
Source: PLoS One. 2025 Jul 17;20(7):e0326032. doi: 10.1371/journal.pone.0326032 (PMC12270128; doi:10.1371/journal.pone.0326032)
Supplement: Attachment — Submitted filename: Reviewer Reply 250512f.docx [file pone.0326032.s004.docx]

Impact of the entorhinal feed-forward connection to the CA3 on hippocampal coding

Response to Reviewers 250512

**Reviewer Comments**

Reviewer #4: In the first round of reviews, I attached a .txt file with all my comments. Apparently, the authors did not see my file. I apologize for not putting everything directly to the authors. I will mention below what was in the first round and any new comments.

The results are interesting, but statistical reporting is lacking, and the manuscript possesses inconsistencies and confounds that should be addressed before suitable for publication.

**Major points**

1. The statistical results are never properly described. In the Figure legends, the authors mention the statistical tests they used, some R-squared values and some figures have the p-values, but never the actual statistics values, confidence intervals, etc. All statistical results should report the statistic test values, degrees of freedom (if applicable), confidence intervals, p values, and a size effect estimate or a model fit. I suggest summarizing all statistical tests in tables that can be in the supplemental material to avoid polluting the main manuscript, but including them in the publication is essential.

**We agree that effect sizes are important. They are now included in results as partial eta-squared (*η*²) values. Appendix tables along with statistic test values, degrees of freedom (if applicable), *p* values, and a partial *η*² estimates or an R^2^ model fit are now added for each figure.**

2. In Line 245, the subtitle "Spike dynamics of feed-forward and feedback axons reveal greater activity in four-tunnel vs. five-tunnel architecture" seems to be in contradiction with the results, and says the opposite of the conclusion in the following paragraph, (line 270) "This means that spike rates were higher overall in the five-tunnel compared to the four-tunnel configuration."

**Line 245 – now says “Dynamics of feed-forward and feedback axons reveal faster spiking in five-tunnel vs. four-tunnel architecture”. This now agrees with lines 271-272 at the end of the paragraph, “slopes in the five-tunnel design were 30-87 % faster than the four-tunnel axon architectures.**

3. In line 296, the authors say they observe the fastest spiking in EC compared to the other subregions, but the graph 3F shows the DG as the fastest.

**We found an error in our 3F bar graph. The current version matches the curves shown in 3A-D. Line 308 now says, “the fastest spiking in EC compared to the other subregions (Fig 3F).”**

4. Data and statistical effects visualization could be much improved in the Figures by completely abandoning barplots. They show no information regarding data distribution and often misinform the distribution in certain scales. Showing the data points or a violin plot along with the mean +- sd/se, for instance, would allow readers to better visualize the data distribution and mean.

**We believe the underlying data distribution is adequately displayed in the log-log cumulative probability distributions described in detail in lines 186-209. The bar plots are a summary of the slopes of the data distribution in the log-log CCF. Data distributions are most useful to validate linear Gaussian distributions about a mean. It would be foolish to do this for 4,000 to 35,000 ISI data points that are log-log distributed over more than an order of magnitude. To see the variation and confidence of the slope from the mean linear fit in the log domain (equation 2 on line 193), we have added R^2^ goodness of fit and other details of the ANCOVA in the supplementary tables.**

**Here is an example of the data distribution and the resultant CCF that results in curve smoothing.**

A) PDF for DG ISI	B) CCF for same DG ISI data

5. From the methods, it is not clear if these neuronal network devices allow for connections not present in the brain (ex., CA3 - EC feedforward connections). If Yes, then

1) The authors should mention it. **Now mentioned in the Limitations section of the Discussion.**

2) Also if yes, this leads to a possibly better experimental control from a network science perspective. In network science, the fact that the 4-tunnel architecture has less routes of interaction could per se account for some of the observed changes here and many changes in most networks (biological or not). Thus an experimental control with the same possible interaction routes would be more appropriate. Although a 5-tunnel architecture with a "wrong connection" is beyond the scope of this study, it could show precisely how, mechanistically, the tri-synaptic design is biologically more efficient. If artificial connections are possible in this method, this possibility of experimental control should be discussed.

**On line 556, we add the limitation in our network “the observation of feedback activity from CA3 to EC (not shown) indicates that some unphysiological connections are also possible when evidence for this connection is lacking in vivo (Ishizuka et al., 1990)**.

Ishizuka N, Weber J, Amaral DG (1990) Organization of intrahippocampal projections originating from CA3 pyramidal cells in the rat. J Comp Neurol 295:580-623.10.1002/cne.902950407

6. The names of the architectures vary in many ways across the whole manuscript (eg. 5-tunnel and 4-tunnel and four-tunnel and five-tunnel) and should be standardized.

**Naming consistency is now four-tunnel and five-tunnel throughout, except for the spatially constrained figures.**

**Minor points**

7. The authors could gain much leverage from using statistical models that better fit their experimental design, such as linear mixed models with random effects on the plate. such models also allow for generalizations to other distributions beyond gaussian (eg. Poisson, gamma, etc).

**We do have categorical differences that we are most interested in……e.g. slopes for the log-log distribution of interspike intervals (ISI) between four-tunnel vs. five-tunnel network architectures. And we used ANCOVA to test whether the two architectures are likely to result from two different populations or whether their variances are likely to result from subsamples of a single distribution. The detailed statistics are now included in supplemental tables.**

8. Authors should update the link where the data and code are available since the given one seems to not exist or has been changed.

**The problem with Dryad, has been** fixed.

9. In SupFig1, panels C-D, if the graphs match the colors of the dots OR the curves to that of the legend, it would help a more rapid identification.

**The curves are now adequately identified by the colors of the R^2^ values and names**

10. The "legend titles" should all be reviewed and corrected. They show several inconsistencies. For example, in Fig 2 the legend is missing the 5-tunnel architecture name. In Fig 5, the legend title says the opposite of what the results say. In Fig 6, the legend title does not seem to make sense.

**Fig 2 now addressed in a revised figure**

**Fig 5 In line 390, we replaced faster with slower**

11. In lines 304-308, "Comparison of the two tunnel types in Fig 3G, shows only a 4% higher spike rate in EC for the five-connect networks, while all the others produced an 11-80% advantage for the four-tunnel networks over those of the five-connect.". However, in Fig 3G, the difference magnitude between the EC bars seems comparable to the largest difference in other subregions (that of CA1).

**Sorry, but we don’t understand “seems comparable to the largest difference in other subregions (that of CA1).” The difference in CA1 appears as an 80% decrease in slope for the five- relative to the four-tunnel, compared to the stated 4% increase for EC.**

12.The bar graphs show p-values and deltas in some figures but not in others containing similar comparisons. Showing those per comparison along with the deltas mentioned in the main text will facilitate matching text and graph and reveal the issue in the previous comment.

**Our figures from the last revision have fixed these problems.**
